# Among-Population Variation in Tolerance to Larval Herbivory by *Anthocharis cardamines* in the Polyploid Herb *Cardamine pratensis*

**DOI:** 10.1371/journal.pone.0099333

**Published:** 2014-06-19

**Authors:** Malin A. E. König, Kari Lehtilä, Christer Wiklund, Johan Ehrlén

**Affiliations:** 1 Department of Ecology, Environment and Plant Sciences, Stockholm University, Stockholm, Sweden; 2 School of Natural Science, Technology and Environmental Studies, Södertörn University, Huddinge, Sweden; 3 Department of Zoology, Stockholm University, Stockholm, Sweden; Oxford Brookes University, United Kingdom

## Abstract

Plants have two principal defense mechanisms to decrease fitness losses to herbivory: tolerance, the ability to compensate fitness after damage, and resistance, the ability to avoid damage. Variation in intensity of herbivory among populations should result in variation in plant defense levels if tolerance and resistance are associated with costs. Yet little is known about how levels of tolerance are related to resistance and attack intensity in the field, and about the costs of tolerance. In this study, we used information about tolerance and resistance against larval herbivory by the butterfly *Anthocharis cardamines* under controlled conditions together with information about damage in the field for a large set of populations of the perennial plant *Cardamine pratensis*. Plant tolerance was estimated in a common garden experiment where plants were subjected to a combination of larval herbivory and clipping. We found no evidence of that the proportion of damage that was caused by larval feeding vs. clipping influenced plant responses. Damage treatments had a negative effect on the three measured fitness components and also resulted in an earlier flowering in the year after the attack. Tolerance was related to attack intensity in the population of origin, i.e. plants from populations with higher attack intensity were more likely to flower in the year following damage. However, we found no evidence of a relationship between tolerance and resistance. These results indicate that herbivory drives the evolution for increased tolerance, and that changes in tolerance are not linked to changes in resistance. We suggest that the simultaneous study of tolerance, attack intensity in the field and resistance constitutes a powerful tool to understand how plant strategies to avoid negative effects of herbivore damage evolve.

## Introduction

Plant fitness may be reduced by herbivores directly, through the consumption of reproductive parts, or indirectly through consumption of vegetative parts impairing resource acquisition [1]–[3]. Tolerance is defined as the ability to reduce plant fitness losses after herbivore attack, e.g. through increased growth rate, assimilation rate, changed morphology or relocation of stored carbon storages [4]–[6]. In the absence of herbivory, investment in high tolerance might reduce the available resources to other functions and plant fitness [7]. The optimal levels of tolerance should therefore depend on the overall intensity of herbivory and the costs of defense [8]. Moreover, the relative advantages of different tolerance mechanisms depend on the type of damage [9], [10]. Variation in the intensity and type of herbivory among populations should thus create a pattern of varying tolerance mechanisms and degree of investment in tolerance as a defense among plant populations [11]. Yet, relatively few studies have investigated differences in tolerance among populations within species, and examined how such differences are related to variations in herbivory intensity. Of these studies, some have found differences in tolerance between populations [12], [13] whilst others have not [14]–[16].

The alternative strategy to reduce fitness losses due to herbivory is resistance, i.e. avoidance of herbivory by either increased plant defenses or through escape from herbivores [17]. Tolerance and resistance are often seen as functionally redundant defense strategies, since tolerant plants should benefit less from avoiding damage, and resistant plants should benefit less from compensating fitness losses due to damage [18], [19]. Compared to tolerance, resistance affects the fitness of the herbivore and may trigger a coevolutionary arms race [20]–[22]. Both strategies are potentially costly in terms of drawing resources from other plant functions [7], [23] and we would thus expect a trade-off between investment in tolerance and resistance [24]. However, if the herbivore is locally adapted to a plant’s resistance mechanism, the effect of resistance drops [22] and instead tolerance might be favored [25]. It is thus important to study the relationship between tolerance, resistance and attack intensity simultaneously in natural populations.

*Cardamine pratensis* is a perennial herb, with both tetraploid and octoploid populations occurring in Sweden [26]. Both ploidy types are used for oviposition by the butterfly *Anthocharis cardamines*, but tetraploid populations are often more attacked than octoploid populations [27]. A previous study with this study system [13] showed that average tolerance levels of populations were positively correlated with attack intensity in the field. The main question addressed in this study is how among-population variation in tolerance is related to variation in both resistance and attack rates among natural populations. Based on the simultaneous information about among-population variation in tolerance under controlled conditions, resistance under controlled conditions and exposure to damage by *A. cardamines* in the field, we then discuss the relative importance of tolerance and resistance for variation in attack rates in natural populations, and the evolution of increased tolerance in general. When measuring plant fitness, we also address the question whether herbivore damage influences if and when plants re-flower in the following year. Because we used a combination of larval feeding and artificial clipping damage to measure tolerance, we test whether larval and artificial damage differ in their effect on *C. pratensis*. We also confirm the findings concerning tolerance in the previous study in the same system [13], using a much more extensive set consisting of 25 tetraploid and 28 octoploid populations.

## Method

### Study System

*Cardamine pratensis* L. (Brassicaceae) is a polyploid complex distributed throughout Europe and Central and Eastern Asia [26]. Tetraploids and octoploids are common in Sweden. Tetraploids are smaller, produce smaller but more abundant flowers, and occur in sunnier environments than octoploids [27]. Both ploidy types are highly clonal and easy to propagate from leaves and leaflets.

*Anthocharis cardamines* L. (Lepidoptera: Pieridae) flies during May-June, and uses several Brassicaceae species as larval host plants, but often shows a strong preference for *C. pratensis* [28]–[30]. The female prefers plants that have just begun to flower [29], and oviposits a single egg per plant in the inflorescence together with an oviposition deterrent pheromone to discourage other females from utilizing the same host plant [31]–[33]. The newly hatched larva initially feeds on the buds, flowers and young siliquae, but often consumes the whole flowering shoot and most of *C. pratensis*’ leaf rosette before pupation [30].

### Study Design

To examine among-population variation in plant tolerance to *A. cardamines* larval attacks, we collected leaf material from 2 to 5 individuals in 53 populations, whereof 25 tetraploid and 28 octoploid populations, located in a 95 km^2^ large area in the parish of Ludgo, Sweden, during the summer 2009. All populations grew on privately owned land, and collection of plant material and field observations were conducted in agreement with the land owners. Multiple replicates (ramets) of each sampled individual (genet) were produced by potting leaflets in sowing soil in the greenhouse. Potted plants were cultivated in a common garden at Stockholm University from August till May. For the tolerance experiment, carried out during the summer 2010, only genets represented by at least two flowering ramets were included. Two to five flowering ramets per genet and two to five genets per population were used. In total 829 plants representing 177 genets from 53 populations were included in the experiment.

The two to five flowering ramets of one genet were randomly assigned to one of two treatments. One to three flowering ramets per genet were oviposited upon with a single egg by *A. cardamines* during a preference experiment (for details on the experimental set up see paragraph *Tolerance, resistance and attack intensity in the field*), and one to two flowering ramets per genet were kept as controls and were not oviposited upon. After oviposition, the plants were placed in the common garden in water-filled trays. The water-filled trays acted as a moat, preventing the larvae from moving between the plants. Within 2–3 weeks after oviposition, the eggs hatched and the larvae began consuming siliques and vegetative parts of their host plants. To plants which were rejected for oviposition in the experiment, we added a first instar larvae hatched in the laboratory. Once every week, each plant was checked for the presence of a larva. At the time when the larva was no longer present, damage was standardized among treated plants by reducing plant mass to 5% of the original biomass through clipping. Allowing larval consumption of plant parts before clipping increases the probability of triggering induced responses to herbivory, thus making plant responses more similar to natural conditions [33]. The choice of a 95% reduction of the original plant material via clipping was based on that 95% was the highest amount of consumed material by larvae in a ramet observed in the study. Damaged and control plants were randomly placed in the common garden and kept over winter. In 2011, survival, flower number, shoot basal diameter and shoot height were recorded for each plant. To investigate if herbivore damage influenced the reproductive phenology, individuals with flower buds were also observed once a week until all plants had begun flowering.

Population means for damaged and control plants within each ploidy type were calculated for three fitness components: proportion of ramets surviving (hereafter survival), proportion of surviving ramets flowering (hereafter probability of flowering), and mean number of flowers in flowering ramets (hereafter number of flowers). *Cardamine pratensis* is self-incompatible and needs to be cross-pollinated by insects [26], e.g. different Empididae species, to produce seeds. However, the two ploidy types are compatible [26] but seed set after pollination between ploidy levels is unknown. Because plants were grown in a common garden setting where pollinators could transfer pollen both among ramets of the same clone and between the two ploidy types, seed set was not regarded as a reliable estimate of fitness since it might reflect mating opportunities rather than the effect of treatment. Instead, we used mean total number of flowers of all individuals (hereafter total flower production) as a measure of total fitness. Non-surviving and non-flowering individuals were assigned a zero for flower number. Total flower production is the product of the three fitness components. In addition, flower shoot mass was used as an indicator of plant size, and was estimated as the volume of a cylinder, where the basal diameter of the inflorescence stem represents the diameter of the cylinder and the height of the inflorescence stem represents the height of the cylinder. Flower shoot mass was log transformed to improve normal distribution. First day of flowering was estimated by the number of days since the first of May until the first flower opened. All statistical analyses were conducted in R 2.15.3, package car [35], [36].

### Plant Responses to Treatment

The effects of treatment, ploidy type and their interaction on the population mean of the three fitness components and total flower production were analyzed using two-way ANOVA. To investigate if phenology and plant size were affected by herbivory the previous year we also ran models with population mean of first day of flowering and population mean of plant size as response variables (Table S1).

Tolerance is commonly estimated by the regression slope of fitness over a damage gradient [5]. We estimated the slope by calculating the difference in fitness between our treated and control plants using: (W(treatment)-W(control))/(0.95-0), where W equals average fitness of damaged and control plants of a specific genet, respectively, and the denominator represents the difference in degree of damage between treated (with 95% above-ground biomass removal) and control plants. We calculated tolerance values for each of the three fitness components, survival, probability of flowering, and number of flowers produced as well as for the total flower production. From the genet tolerance means we calculated the population tolerance mean for each tolerance estimate to alleviate the potential problems associated with a low number of ramets sampled per genets (Table S2). All analyses were thus done at the among population level.

By standardizing damage through clipping, we reduced problems associated with differences in larval consumption rates when estimating tolerance. Still, consumption of plant tissue by larvae may influence plants differently from damage by clipping [34], [37], [38]. For example prolonged exposure to larval saliva may trigger induced responses [39]. In our study, the proportion of plant tissue consumed by larvae ranged from 16 to 69% among populations (Table S2). To examine if the proportion of plant tissue consumed by larvae vs. clipping influenced plant responses and tolerance we used linear models to test the effects of the proportion of plant tissues consumed by larvae and ploidy type on each of the three tolerance estimates.

### Tolerance, Resistance and Attack Intensity in the Field

To examine the relationship between tolerance, resistance and attack intensities in the field we used estimates of resistance and attack rate from a previous study with the same study system [40]. To estimate attack intensities in the field, we searched up to 30 flowering individuals for eggs during the flowering seasons 2009–2011 in 21 of the 53 populations (ten tetraploid and eleven octoploid) included in the tolerance experiment. The attack intensity within each population was estimated as the proportion of plants that were oviposited upon. To account for between-year variations in butterfly frequency, we relativized attack intensity by subtracting the yearly mean of all populations and then averaging the attack intensity across years.

Plant resistance against oviposition was estimated by placing eight tetraploid and eight octoploid ramets of randomly chosen genets in a cage with a mated female *A. cardamines*. Plants were removed from the cage as soon as they had received an egg. Each cage trial was continued until all 16 plants were oviposited upon or the sun began to descend. If a plant did not become oviposited upon during this time, it was assigned an oviposition time corresponding to the duration of the trial. However, if more than 10 individuals were not oviposited upon during the first day of an experimenat trial, it was continued the following day. Plant resistance was estimated as the time interval from the start of the experiment until a plant was oviposited upon. To be able to compare the resistance estimates between the different cage trials, the oviposition time for each experimental trial was standardized by subtracting the mean oviposition time of the trial and dividing the difference by the standard deviation. This standardized time was used as an estimate of plant resistance for each plant.

We first used four ANCOVA models to test if any of the different tolerance estimates were correlated with resistance, analyzing each ploidy type separately (Table S2). Second, we tested if the four tolerance estimates were related to attack rate and ploidy type (Table S2). Thirdly, we tested if resistance was correlated with attack intensity in the field, analyzing each ploidy type separately (Table S2).

Lastly, we tested if tolerance was negatively correlated to fitness in the absence of herbivory by correlating genet mean tolerance with total flower production in control plants of the same genet. To avoid spurious correlations, multiple ramets from the control group belonging to the same genet were randomly assigned to one of two groups. One group was used as a reference in calculations of tolerance components, using the same formula as above, and the other group was used to calculate fitness in terms of flower production in the absence of damage. Each tolerance value was first calculated using genet means and the genets means were then used to calculate the population mean. Tetraploid and octoploid populations were examined separately.

## Results

### Plant Responses to Treatment

**Figure 1:**
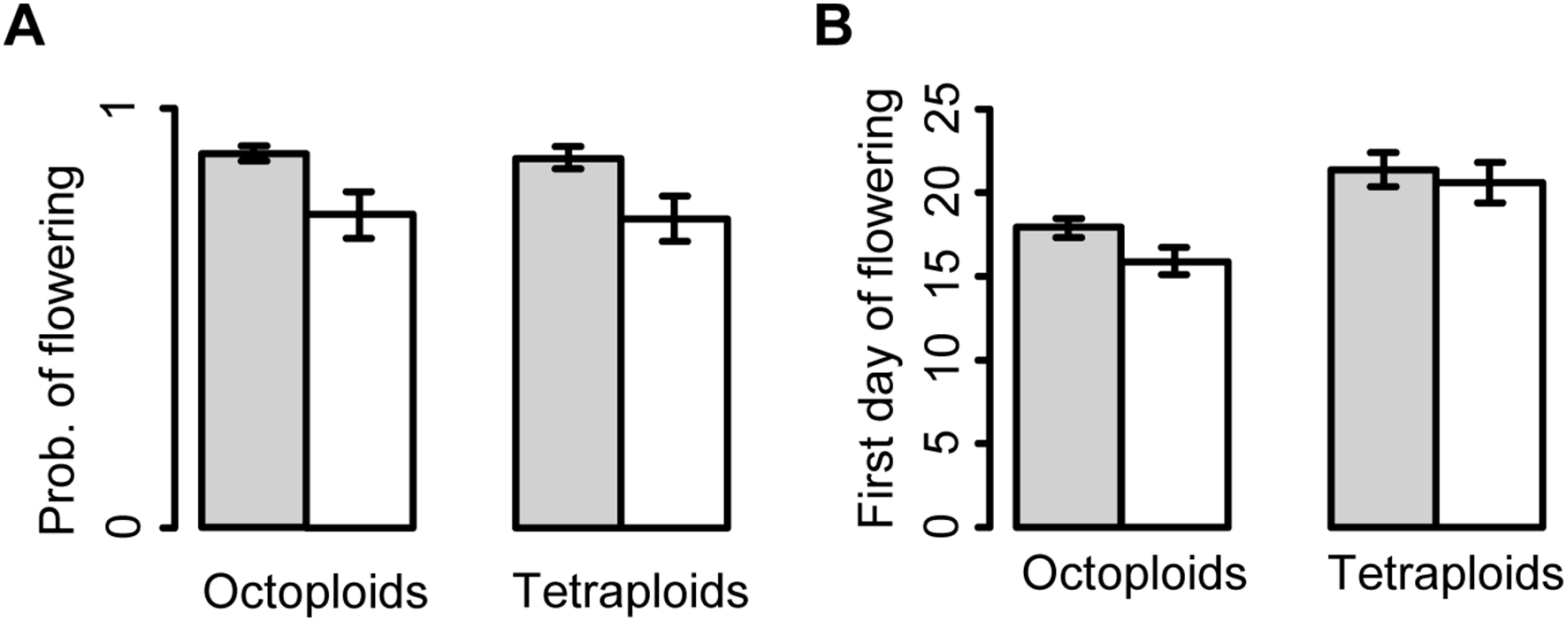
Effects of experimental damage on probability of flowering and phenology in *Cardamine pratensis*. The herbivore damage treatment was a combination of feeding by the larvae of the butterfly *Anthocharis cardamines* and clipping. All responses were recorded the year after the damage treatment. The bars show mean values for 25 tetraploid and 28 octoploid populations for six responses: a. probability to flower and b. first day of flowering. Grey bars are responses for undamaged plants, and open bars are damaged plants. First day of flowering was equal to number of days from first of May until first open flower. Error bars represent±1SE.

Damaged plants were less likely to flower the year after damage (Table 1, Fig. 1a). Interestingly, damaged plants flowered earlier than control plants in the year after damage (Fig. 1b). The effect of treatment on first day of flowering was still significant when number of flowers was included in the model (*F*_1,102_ = 35.3, *P*<0.001). In agreement with previous studies, plants subjected to the damage treatment were less likely to survive and also produced fewer flowers compared to undamaged control plants (Table 1). We did not find significant relationships between any of the four tolerance estimates and the proportion of plant tissue consumed before the plant mass was reduced to 5% through clipping (Table 2). Neither did the four tolerance estimates differ between ploidy types (Table 2).

**Table 1:** The effects of experimental damage and ploidy type on six measures of plant performance in the perennial herb *Cardamine pratensis* the year after the treatment.

	Treatment			Ploidy type			Treatment×Ploidy type		
	df	*F*	*P*	df	*F*	*P*	df	*F*	*P*
Survival	1	4.00	0.049	1	3.55	0.062	1	3.55	0.062
Probability of flowering	1	44.71	<0.001	1	0.16	0.691	1	0.04	0.848
Number of flowers	1	25.61	<0.001	1	119.75	<0.001	1	0.61	0.438
Total flower production	1	48.23	<0.001	1	84.31	<0.001	1	1.83	0.179
First day of flowering	1	9.92	0.002	1	78.23	<0.001	1	2.05	0.155
Flower shoot mass	1	1.32	0.254	1	39.07	<0.001	1	1.86	0.356

Results are for treatments (control and experimental damage) applied to plants from 25 tetraploid and 28 octoploid populations. All responses are population means.

**Table 2:** Relationship between the proportion percentages of plant tissue consumed by the developing larvae and four estimates of plant tolerance in two ploidy types of *Cardamine pratensis*.

Plant tolerance	Percentage plant tissue consumed			Ploidy type		
	df	*F*	*P*	df	*F*	*P*
Survival	1	0.13	0.72	1	2.20	0.14
Probability of flowering	1	0.81	0.37	1	0.07	0.79
Number of flowers	1	0.31	0.58	1	0.16	0.69
Total flower production	1	0.004	0.95	1	2.78	0.10

Tolerance estimates are population means from common garden experiments with plants from 25 tetraploid and 28 octoploid populations. All plants had 95% of their above-ground tissues removed and plants that were less damaged by larval feeding were subjected to also a clipping treatment.

### Tolerance, Resistance and Attack Intensity in the Field

There was no cost of tolerance in terms of negative associations between any of the four tolerance estimates and fitness in the absence of herbivory (*P*>0.05).

**Figure 2:**
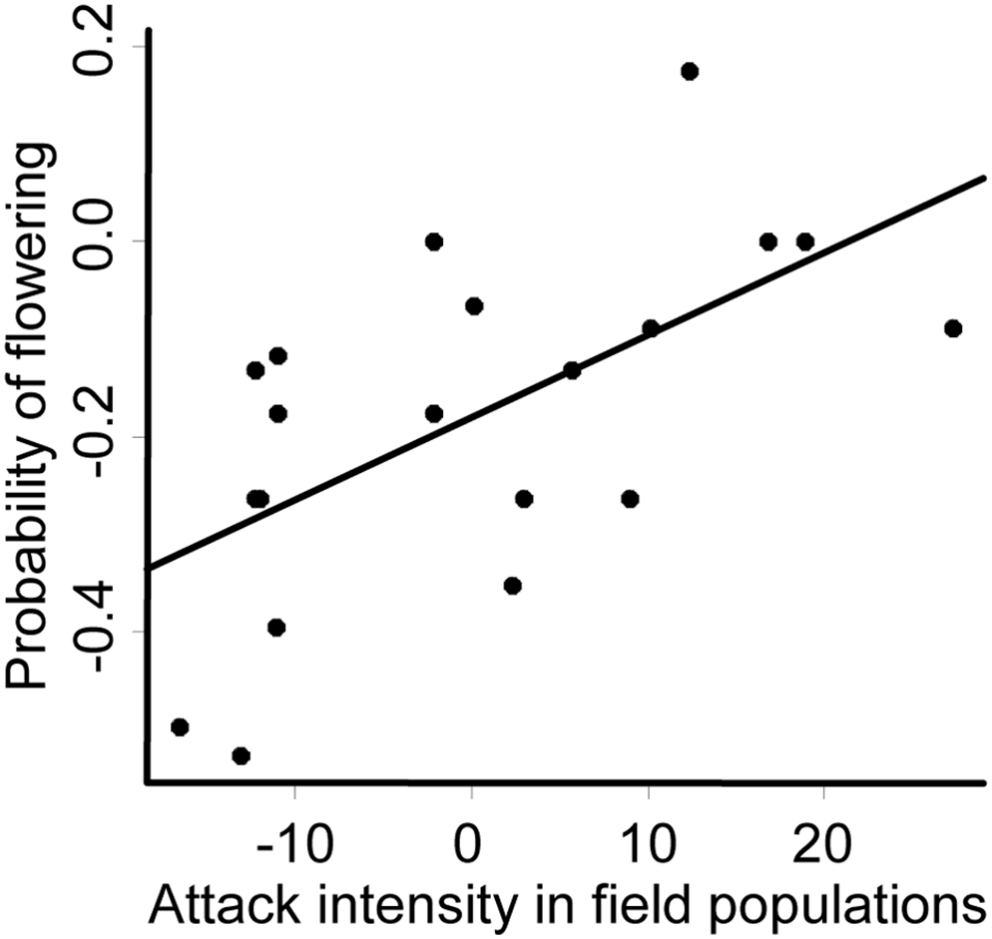
Relationship between tolerance and attack intensity in the field for *Cardamine pratensis*. Tolerance was estimated as the probability of flowering the year after herbivory. Attack intensity was measured as the percentage of oviposited plants in the field populations. Negative values correspond to relatively low attack intensity and positive values to relatively high attack intensity. Each symbol represents the mean values of one population.

Populations that were more exposed to attack by *A. cardamines* were more tolerant, in terms of having higher probability to flower the year after an attack, than plants from populations exposed to lower intensities of attack (Fig. 2, Table 3). We found no relationship, positive or negative, between resistance and the different measures of tolerance in tetraploids or octoploids (Table 4). There was no correlation between the resistance estimates of populations from the cage experiments and attack intensity in natural populations for tetraploids (*r* = 0.36, *n* = 10, *P* = 0.31) or octoploids (*r* = −0.43, *n* = 11, *P* = 0.19).

**Table 3:** Relationship between four estimates of plant tolerance to herbivory and attack intensity in the field and ploidy type in the herb *Cardamine pratensis*.

Plant tolerance	Attack intensity			Ploidy type		
	df	*F*	*P*	df	*F*	*P*
Survival	1	1.72	0.206	1	1.69	0.210
Probability of flowering	1	10.61	0.004	1	0.06	0.804
Number of flowers	1	0.047	0.831	1	0.99	0.332
Total flower production	1	1.62	0.220	1	7.37	0.014

Tolerance estimates are from common garden experiments using population means from 10 tetraploid and 11 octoploid populations.

**Table 4:** Relationship between four estimates of tolerance and variation in resistance among populations of two ploidy types in the perennial herb *Cardamine pratensis*.

Plant tolerance	Plant resistance			
	Tetraploids		Octoploids	
	*r*	*P*	*r*	*P*
Survival	NA	NA	−0.19	0.328
Probability of flowering	−0.37	0.069	0.28	0.152
Number of flowers	0.14	0.497	0.10	0.608
Total flower production	−0.03	0.880	0.32	0.095

Values are Pearson’s correlation coefficients calculated using means of 25 tetraploid and 28 octoploid populations. Plant resistance against oviposition and the four estimates of tolerance were estimated under controlled environmental conditions (see Methods for explanation). Because all plants survived, the relationship between survival tolerance and plant resistance against oviposition for tetraploids is not included.

## Discussion

Our results show that damage both reduced the probability of flowering and altered the flowering phenology the year after treatment. Plant responses did not differ between larval feeding and clipping. Tolerance to damage was not correlated with plant resistance against oviposition estimated under controlled conditions. However, populations with higher attack intensities by *A. cardamines* in the field were more tolerant to larval herbivory under controlled conditions than populations experiencing low attack intensity [13]. We also confirmed that tolerance was not related to any costs in the absence of herbivory [13].

Damage had a negative effect on all investigated fitness components. Damage restricted to reproductive parts might result in lower reproductive effort and increased allocation to vegetative growth and clonal reproduction, thereby decreasing the strength of selection on plant defenses. However, an attack by A. cardamines in the field often removes not only reproductive parts but also much of the vegetative tissues, resulting in that also resource acquisition is impaired. In our experiment, 95% of above ground tissue were removed. It thus seems reasonable to assume that observed effects in the experiment correspond to decreases in fitness after damage and that herbivory by A. cardamines constitutes an important selective agent in this system.

In our study, we chose to use a natural damage to trigger potential responses induced by larval chewing, combined with artificial damage to equalize the damage levels. Although artificial and natural damage often have similar effects on plant performance [34], studies have also shown that not all plants respond similarly to artificial damage compared to damage caused by herbivores [37], [38]. This could for example be due to the lack of induced plants responses caused by herbivore saliva [39] or the timing of damage [41]. Herbivore induced plant responses could either increase the longer the larva spends feeding, or the plant could have a time lag before it responds. Since our plants experienced different amounts of larval herbivory before clipping this should have influenced plant responses if type of damage is important. However, we found no correlation between the percentage plant material consumed before clipping and the estimated tolerance. This strongly suggests that *C. pratensis* tolerance responds similarly to artificial and natural damage.

A new finding in this study was that herbivory affected the first day of flowering: damaged plants flowered earlier than non-damaged. This was unexpected since early flowering individuals are more likely to become oviposited upon by *A. cardamines* [30]. Hence, the phenological response of plants to damage should imply that they are more likely to become damaged also in the subsequent year. A reasonable assumption is therefore that the response in flowering phenology is not adaptive in itself, but an indirect effect of developmental processes related to compensation for the resource losses caused by herbivory. Changes in phenology as a result of herbivory are believed to be widespread among plants, but are rarely studied [6], [42]. To our knowledge, no previous studies have experimentally demonstrated that plants respond to herbivory by flowering earlier in the year after the attack.

Tolerance and resistance are traditionally believed to be partly redundant strategies. However, we did not find evidence for a trade-off between tolerance and oviposition resistance, indicating that the two strategies are not necessarily redundant or that there is no allocation cost for the plant to invest in the two strategies simultaneously. The results of previous studies are mixed; some have found tolerance and resistance to be negatively correlated [24], [43] whilst others did not find evidence of a trade-off [8], [44]. More recently, it has been argued that tolerance and resistance are not redundant but complementary strategies [6], especially in systems where plants are exposed to multiple herbivores [45]. Tolerance and resistance could be complementary strategies in *C. pratensis* since it is attacked by herbivores other than *A. cardamines*. For example, heavy attacks by *Phyllotreta* sp. and *Gastrophysa viridula* (Chrysomelidae) have been observed in both ploidy types in some years (pers. obs.).

To evaluate associations and causal relationships between tolerance, attack intensity in the field and resistance and to assess their relative importance in natural populations it is necessary to simultaneously investigate all three parameters. Attack intensity in the field and tolerance are expected to be positively correlated since plants experiencing a higher risk of attack are under stronger selection to reduce the negative effects of herbivore damage on fitness [46]. This study, as well as a previous one with the same study system [13], indeed found a positive relationship between tolerance and attack intensity. Resistance could be a causal factor behind this relationship. First, resistance can be negatively associated with both tolerance and attack intensity. However, in this study we found no negative association between resistance and tolerance or between resistance and attack intensity among populations. Second, although tolerance and resistance traditionally have been claimed to be alternative strategies against herbivory [19], a growing number of studies have found a positive association between tolerance and resistance [8], [47], [48]. If resistance is positively associated with tolerance, the herbivore is restricted to certain populations due to habitat cues and increased attack intensity selects for resistance only, then we would still expect to find a positive spurious association between attack intensity and tolerance. This scenario does not seem likely for our study system because there was no association between tolerance and resistance among populations or between resistance and attack rate in the field. Based on these results, we conclude that the relationship between attack intensity and tolerance in our system is not mediated by resistance, but likely the effect of an increased selection for tolerance in populations exposed to higher intensities of damage in the field. Other studies investigating tolerance levels among populations with different levels of herbivory intensity have found an association between herbivory intensity and tolerance in some cases [12], [13] but not in other [14]–[16]. Few studies have assessed tolerance and resistance under controlled conditions and correlated these results to the attack intensity in natural populations. Tiffin & Rausher [49] examined the relationship between tolerance, resistance and attack rates under natural conditions and found that both selection and the relationship between tolerance and resistance varied depending on the type of damage caused by the herbivore. Bustos-Segura et al. [25] showed that herbivores that are locally adapted to the host plant resistance mechanisms selected for increased tolerance rather than for increased of resistance because the cost of further increases in resistance were greater than the fitness gained [22]. The main defense mechanism of *C. pratensis* is based on glucosinolates and *A. cardamines* is a glucosinolate specialist [50]. The increased tolerance levels observed in our study may thus be due to a highly adapted herbivore. Taken together, this further stresses the importance of simultaneously incorporating experimental assessments of tolerance and resistance and field recordings of intensity of herbivory to better understand the factors governing the evolution of plant defense strategies.

To conclude, our results strongly suggest that herbivory is able to drive the evolution of tolerance without affecting resistance levels. We show that our approach to simultaneously study tolerance, resistance and attack intensity in the field constitutes a powerful tool to understand the ecological settings that promote the evolution of different defensive strategies to minimize the negative effects of herbivore damage.

## Supporting Information

Table S1 **Data on the effects of experimental damage and ploidy type on six measures of plant performance in the perennial herb** ***Cardamine pratensis*** **the year after the treatment.**
(DOCX)

Table S2 **Data on the population mean of the four tolerance estimates in** ***Cardamine pratensis*** **, the population mean of proportion of plant material consumed by an** ***Anthocharis cardamines*** **larva before clipping, the population mean resistance estimated under controlled conditions and the population mean attack intensity in the population of origin.**
(DOCX)
