# Supplementary material for: Among-Population Variation in Tolerance to Larval Herbivory by Anthocharis cardamines in the Polyploid Herb Cardamine pratensis
Source: PLoS One. 2014 Jun 19;9(6):e99333. doi: 10.1371/journal.pone.0099333 (PMC4063699; doi:10.1371/journal.pone.0099333)
Supplement: Table S1 — Data on the effects of experimental damage and ploidy type on six measures of plant performance in the perennial herb Cardamine pratensis the year after the treatment. (DOCX) [file pone.0099333.s001.docx]

**Table S1.** Data on the effects of experimental damage and ploidy type on six measures of plant performance in the perennial herb *Cardamine pratensis* the year after the treatment from 53 populations.

| Population | Treatment | Ploidy type | Survival | Probability of flowering | Number of flowers | Total flower production | First day of flowering | Flower shoot mass |
| --- | --- | --- | --- | --- | --- | --- | --- | --- |
| Almvik | Damaged | Octoploid | 0.889 | 0.625 | 12.000 | 6.667 | 15.122 | -0.485 |
| Aspa2 | Damaged | Octoploid | 1.000 | 0.667 | 6.333 | 4.222 | 18.738 | -0.957 |
| AspaIP2 | Damaged | Octoploid | 1.000 | 0.857 | 7.500 | 6.429 | 16.178 | -0.620 |
| Bysjön | Damaged | Octoploid | 1.000 | 0.923 | 6.917 | 6.385 | 16.835 | -0.497 |
| Bölsäter1 | Damaged | Octoploid | 1.000 | 1.000 | 11.333 | 11.333 | 15.604 | -0.549 |
| Dammen | Damaged | Octoploid | 1.000 | 0.500 | 3.667 | 1.833 | 21.067 | -0.596 |
| Davik | Damaged | Octoploid | 1.000 | 1.000 | 13.750 | 13.750 | 12.750 | -0.415 |
| Djupbrodal | Damaged | Octoploid | 1.000 | 1.000 | 8.444 | 8.444 | 13.011 | -0.583 |
| Edeby2 | Damaged | Octoploid | 1.000 | 0.909 | 8.900 | 8.091 | 12.300 | -0.346 |
| Gustav | Damaged | Octoploid | 0.875 | 0.857 | 11.833 | 9.300 | 15.510 | -0.368 |
| Horssjön | Damaged | Octoploid | 0.917 | 0.545 | 6.667 | 3.333 | 18.775 | -0.403 |
| Inskogsbergen | Damaged | Octoploid | 1.000 | 0.800 | 10.500 | 8.400 | 15.179 | -0.621 |
| Klippan | Damaged | Octoploid | 1.000 | 0.778 | 9.000 | 7.000 | 16.836 | -0.333 |
| Kryckeläng | Damaged | Octoploid | 1.000 | 0.700 | 6.714 | 4.700 | 16.040 | -0.508 |
| Larslund1 | Damaged | Octoploid | 1.000 | 0.909 | 10.900 | 9.909 | 14.878 | -0.415 |
| Larslund4 | Damaged | Octoploid | 1.000 | 0.909 | 8.400 | 7.636 | 15.392 | -0.554 |
| Långbro | Damaged | Octoploid | 1.000 | 0.500 | 8.000 | 4.000 | 13.289 | -0.525 |
| N. Ämtvik1 | Damaged | Octoploid | 1.000 | 0.909 | 9.500 | 8.636 | 15.874 | -0.298 |
| N. Ämtvik2 | Damaged | Octoploid | 1.000 | 0.909 | 12.000 | 10.909 | 15.442 | -0.327 |
| N. Ämtvik3 | Damaged | Octoploid | 1.000 | 0.875 | 5.286 | 4.625 | 18.817 | -0.679 |
| Nilslund1 | Damaged | Octoploid | 1.000 | 0.571 | 13.250 | 7.571 | 18.350 | -0.322 |
| Nilslund2 | Damaged | Octoploid | 1.000 | 0.769 | 12.800 | 9.846 | 14.433 | -0.196 |
| Norska1 | Damaged | Octoploid | 1.000 | 0.833 | 10.000 | 8.333 | 14.922 | -0.092 |
| Rågången | Damaged | Octoploid | 0.800 | 1.000 | 14.750 | 11.800 | 14.006 | -0.260 |
| Rågången2 | Damaged | Octoploid | 1.000 | 1.000 | 10.200 | 10.200 | 14.533 | -0.245 |
| Svarv | Damaged | Octoploid | 1.000 | 0.833 | 4.900 | 4.083 | 17.370 | -0.706 |
| V-m2 | Damaged | Octoploid | 1.000 | 1.000 | 5.500 | 5.500 | 19.092 | -0.550 |
| Ö. Ämtvik2 | Damaged | Octoploid | 1.000 | 1.000 | 7.833 | 7.833 | 13.903 | -0.628 |
| Almvik | Controll | Octoploid | 1.000 | 1.000 | 14.500 | 14.500 | 16.065 | -0.437 |
| Aspa2 | Controll | Octoploid | 1.000 | 1.000 | 16.000 | 16.000 | 17.485 | -0.341 |
| AspaIP2 | Controll | Octoploid | 1.000 | 1.000 | 9.000 | 7.500 | 16.390 | -0.587 |
| Bysjön | Controll | Octoploid | 1.000 | 1.000 | 9.750 | 9.750 | 19.313 | -0.430 |
| Bölsäter1 | Controll | Octoploid | 1.000 | 1.000 | 14.571 | 14.571 | 19.221 | -0.556 |
| Dammen | Controll | Octoploid | 1.000 | 0.875 | 6.714 | 5.875 | 20.886 | -0.538 |
| Davik | Controll | Octoploid | 1.000 | 1.000 | 15.333 | 14.375 | 17.170 | -0.926 |
| Djupbrodal | Controll | Octoploid | 1.000 | 0.857 | 13.667 | 11.714 | 15.485 | -0.398 |
| Edeby2 | Controll | Octoploid | 1.000 | 1.000 | 10.167 | 10.167 | 15.101 | -0.383 |
| Gustav | Controll | Octoploid | 1.000 | 1.000 | 9.500 | 9.500 | 16.735 | -0.256 |
| Horssjön | Controll | Octoploid | 1.000 | 0.750 | 13.333 | 10.000 | 18.202 | -0.509 |
| Inskogsbergen | Controll | Octoploid | 1.000 | 0.857 | 11.667 | 10.000 | 17.873 | -0.446 |
| Klippan | Controll | Octoploid | 1.000 | 1.000 | 18.500 | 18.500 | 17.858 | -0.308 |
| Kryckeläng | Controll | Octoploid | 1.000 | 1.000 | 14.286 | 14.286 | 17.964 | -0.377 |
| Larslund1 | Controll | Octoploid | 1.000 | 1.000 | 15.571 | 15.571 | 17.942 | -0.391 |
| Larslund4 | Controll | Octoploid | 1.000 | 1.000 | 15.375 | 15.375 | 17.892 | -0.454 |
| Långbro | Controll | Octoploid | 1.000 | 1.000 | 9.500 | 9.500 | 17.676 | -0.500 |
| N. Ämtvik1 | Controll | Octoploid | 1.000 | 1.000 | 15.625 | 15.625 | 16.366 | -0.546 |
| N. Ämtvik2 | Controll | Octoploid | 1.000 | 1.000 | 14.750 | 14.750 | 18.619 | -0.256 |
| N. Ämtvik3 | Controll | Octoploid | 1.000 | 1.000 | 10.000 | 10.000 | 17.564 | -0.592 |
| Nilslund1 | Controll | Octoploid | 1.000 | 1.000 | 11.600 | 11.714 | 20.438 | -0.261 |
| Nilslund2 | Controll | Octoploid | 1.000 | 1.000 | 14.250 | 14.250 | 17.934 | -0.128 |
| Norska1 | Controll | Octoploid | 1.000 | 1.000 | 15.375 | 15.375 | 17.495 | -0.287 |
| Rågången | Controll | Octoploid | 1.000 | 1.000 | 12.000 | 12.000 | 16.583 | -0.137 |
| Rågången2 | Controll | Octoploid | 1.000 | 1.000 | 16.250 | 16.250 | 17.608 | -0.283 |
| Svarv | Controll | Octoploid | 1.000 | 1.000 | 6.875 | 6.875 | 18.213 | -0.838 |
| V-m2 | Controll | Octoploid | 1.000 | 1.000 | 8.500 | 8.500 | 22.393 | -1.200 |
| Ö. Ämtvik2 | Controll | Octoploid | 1.000 | 1.000 | 6.000 | 6.000 | 20.068 | -0.422 |
| Bogslund | Damaged | Tetraploid | 1.000 | 0.750 | 14.222 | 10.667 | 23.959 | -0.619 |
| Bölsäter2 | Damaged | Tetraploid | 1.000 | 0.750 | 18.667 | 12.889 | 19.325 | -0.609 |
| Bölsäter3 | Damaged | Tetraploid | 1.000 | 0.857 | 13.333 | 11.429 | 22.511 | -0.661 |
| Dagnäs1 | Damaged | Tetraploid | 1.000 | 0.917 | 17.636 | 16.167 | 18.590 | -0.562 |
| Dagnäs2 | Damaged | Tetraploid | 1.000 | 1.000 | 20.333 | 20.333 | 20.022 | -0.714 |
| Edeby1 | Damaged | Tetraploid | 1.000 | 0.833 | 16.600 | 13.833 | 20.229 | -0.752 |
| Gravfältet | Damaged | Tetraploid | 1.000 | 0.818 | 22.667 | 18.545 | 18.191 | -0.607 |
| Grinda | Damaged | Tetraploid | 1.000 | 0.875 | 13.429 | 11.750 | 16.529 | -0.730 |
| Kallmyra1 | Damaged | Tetraploid | 1.000 | 1.000 | 12.857 | 12.857 | 25.693 | -0.904 |
| Kallmyra2 | Damaged | Tetraploid | 1.000 | 0.818 | 19.444 | 15.909 | 18.971 | -0.629 |
| Kallmyra3 | Damaged | Tetraploid | 1.000 | 0.917 | 16.364 | 15.000 | 21.699 | -0.866 |
| Kohagen | Damaged | Tetraploid | 1.000 | 0.583 | 17.000 | 9.917 | 26.158 | -0.787 |
| Kristinelund1 | Damaged | Tetraploid | 1.000 | 1.000 | 16.333 | 16.556 | 19.310 | -0.542 |
| Kristinelund6 | Damaged | Tetraploid | 1.000 | 1.000 | 25.333 | 25.333 | 13.914 | -0.481 |
| Larslund2 | Damaged | Tetraploid | 1.000 | 0.909 | 17.600 | 16.000 | 20.979 | -0.600 |
| Larslund3 | Damaged | Tetraploid | 1.000 | 0.889 | 18.750 | 17.545 | 16.088 | -0.719 |
| Norska2 | Damaged | Tetraploid | 1.000 | 0.625 | 14.600 | 10.000 | 25.564 | -0.977 |
| Ryssinge1 | Damaged | Tetraploid | 1.000 | 0.667 | 12.500 | 8.333 | 22.465 | -1.256 |
| Ryssinge2 | Damaged | Tetraploid | 1.000 | 0.556 | 16.200 | 9.889 | 20.861 | -0.682 |
| Ryssinge3 | Damaged | Tetraploid | 1.000 | 0.923 | 15.667 | 14.462 | 19.316 | -0.883 |
| Skogstorp 6ny | Damaged | Tetraploid | 1.000 | 0.800 | 15.750 | 12.600 | 19.764 | -0.771 |
| Skogstorp1 | Damaged | Tetraploid | 1.000 | 0.714 | 12.800 | 8.222 | 23.220 | -0.857 |
| Stene | Damaged | Tetraploid | 1.000 | 0.800 | 23.250 | 17.500 | 17.970 | -0.532 |
| Vildhägn1 | Damaged | Tetraploid | 1.000 | 0.500 | 17.333 | 8.667 | 22.000 | -0.999 |
| V-m1 | Damaged | Tetraploid | 1.000 | 0.857 | 13.583 | 11.643 | 22.307 | -0.822 |
| Bogslund | Controll | Tetraploid | 1.000 | 1.000 | 16.714 | 16.714 | 24.705 | -0.863 |
| Bölsäter2 | Controll | Tetraploid | 1.000 | 1.000 | 22.800 | 22.800 | 19.731 | -0.675 |
| Bölsäter3 | Controll | Tetraploid | 1.000 | 1.000 | 17.200 | 17.200 | 20.050 | -0.767 |
| Dagnäs1 | Controll | Tetraploid | 1.000 | 1.000 | 24.750 | 24.750 | 18.750 | -0.555 |
| Dagnäs2 | Controll | Tetraploid | 1.000 | 1.000 | 23.167 | 23.167 | 20.004 | -0.497 |
| Edeby1 | Controll | Tetraploid | 1.000 | 1.000 | 22.875 | 22.875 | 21.608 | -0.549 |
| Gravfältet | Controll | Tetraploid | 1.000 | 0.750 | 27.167 | 20.375 | 22.429 | -0.455 |
| Grinda | Controll | Tetraploid | 1.000 | 1.000 | 11.333 | 11.333 | 28.567 | -1.067 |
| Kallmyra1 | Controll | Tetraploid | 1.000 | 0.833 | 22.400 | 18.667 | 21.200 | -0.614 |
| Kallmyra2 | Controll | Tetraploid | 1.000 | 1.000 | 32.000 | 32.000 | 20.047 | -0.280 |
| Kallmyra3 | Controll | Tetraploid | 1.000 | 1.000 | 22.375 | 22.375 | 22.513 | -0.603 |
| Kohagen | Controll | Tetraploid | 1.000 | 0.875 | 18.714 | 16.375 | 27.427 | -0.762 |
| Kristinelund1 | Controll | Tetraploid | 1.000 | 1.000 | 33.500 | 33.500 | 17.693 | -0.549 |
| Kristinelund6 | Controll | Tetraploid | 1.000 | 1.000 | 29.000 | 29.000 | 19.750 | -0.240 |
| Larslund2 | Controll | Tetraploid | 1.000 | 1.000 | 16.286 | 16.286 | 20.339 | -0.804 |
| Larslund3 | Controll | Tetraploid | 1.000 | 1.000 | 25.000 | 25.000 | 20.782 | -0.718 |
| Norska2 | Controll | Tetraploid | 1.000 | 0.833 | 17.400 | 14.500 | 21.920 | -0.822 |
| Ryssinge1 | Controll | Tetraploid | 1.000 | 1.000 | 11.800 | 11.800 | 21.250 | -0.956 |
| Ryssinge2 | Controll | Tetraploid | 1.000 | 1.000 | 19.500 | 19.500 | 23.736 | -0.777 |
| Ryssinge3 | Controll | Tetraploid | 1.000 | 1.000 | 19.375 | 19.375 | 21.754 | -0.531 |
| Skogstorp 6ny | Controll | Tetraploid | 1.000 | 1.000 | 15.500 | 15.500 | 22.600 | -0.612 |
| Skogstorp1 | Controll | Tetraploid | 1.000 | 1.000 | 21.400 | 21.400 | 19.591 | -0.884 |
| Stene | Controll | Tetraploid | 1.000 | 1.000 | 21.000 | 21.000 | 18.733 | -0.615 |
| Vildhägn1 | Controll | Tetraploid | 1.000 | 1.000 | 16.750 | 13.400 | 18.292 | -0.695 |
| V-m1 | Controll | Tetraploid | 1.000 | 1.000 | 25.750 | 25.750 | 21.021 | -0.631 |

Results are for treatments (undamaged and experimentally damaged) applied to plants from 25 tetraploid and 28 octoploid populations. All numerical values represent population means.
