# Supplementary material for: Among-Population Variation in Tolerance to Larval Herbivory by Anthocharis cardamines in the Polyploid Herb Cardamine pratensis
Source: PLoS One. 2014 Jun 19;9(6):e99333. doi: 10.1371/journal.pone.0099333 (PMC4063699; doi:10.1371/journal.pone.0099333)
Supplement: Table S2 — Data on the population mean of the four tolerance estimates in Cardamine pratensis , the population mean of proportion of plant material consumed by an Anthocharis cardamines larva before clipping, the population mean resistance estimated under controlled conditions and the population mean attack intensity in the population of origin. (DOCX) [file pone.0099333.s002.docx]

**Table S2.** Data on population mean of four tolerance estimates in *Cardamine pratensis*, population mean percentage plant tissues consumed by *Anthocharis cardamines* larva before clipping, population mean resistance estimated under controlled conditions, and population mean attack intensity in the population of origin.

| Population | Ploidy type | Percentage plant tissue consumed | Resistance | Attack intensity | Survival | Probability of flowering | Number of flowers | Total flower production |
| --- | --- | --- | --- | --- | --- | --- | --- | --- |
| Almvik | Octoploid | 19.556 | -0.420 |  | -0.117 | -0.409 | -0.526 | -8.246 |
| Aspa2 | Octoploid | 46.667 | -0.580 |  | 0.000 | -0.482 | -7.193 | -12.939 |
| AspaIP2 | Octoploid | 39.714 | 0.120 |  | 0.000 | -0.175 | -1.053 | -2.105 |
| Bysjön | Octoploid | 15.958 | -0.287 | -16.761 | 0.000 | -0.088 | -2.807 | -3.465 |
| Bölsäter1 | Octoploid | 54.636 | 0.090 | 11.119 | 0.000 | 0.000 | -1.469 | -1.469 |
| Dammen | Octoploid | 30.458 | -0.163 | 12.312 | 0.000 | -0.395 | -3.289 | -4.254 |
| Davik | Octoploid | 42.375 | 0.293 | 11.952 | 0.000 | 0.000 | -1.988 | -1.988 |
| Djupbrodal | Octoploid | 27.278 | -0.030 |  | 0.000 | 0.000 | -5.497 | -5.497 |
| Edeby2 | Octoploid | 38.182 | -0.180 |  | 0.000 | -0.088 | -1.754 | -2.763 |
| Gustav | Octoploid | 16.222 | -0.444 | 2.128 | -0.117 | -0.117 | 3.509 | 1.053 |
| Horssjön | Octoploid | 22.125 | 0.000 | 11.017 | -0.088 | -0.263 | -6.842 | -7.018 |
| Inskogsbergen | Octoploid | 41.100 | -0.450 |  | 0.000 | -0.219 | -0.702 | -3.026 |
| Klippan | Octoploid | 54.444 | 0.020 |  | 0.000 | -0.234 | -10.468 | -12.105 |
| Kryckeläng | Octoploid | 25.350 | -0.583 | -2.914 | 0.000 | -0.263 | -9.386 | -11.009 |
| Larslund1 | Octoploid | 30.591 | -0.170 |  | 0.000 | -0.088 | -5.307 | -6.360 |
| Larslund4 | Octoploid | 42.318 | -0.500 |  | 0.000 | -0.066 | -8.289 | -8.816 |
| Långbro | Octoploid | 33.042 | -0.244 | -10.094 | 0.000 | -0.526 | -5.439 | -5.789 |
| N. Ämtvik1 | Octoploid | 40.227 | -0.800 |  | 0.000 | -0.088 | -6.491 | -7.061 |
| N. Ämtvik2 | Octoploid | 42.955 | -0.360 |  | 0.000 | -0.088 | -3.640 | -4.298 |
| N. Ämtvik3 | Octoploid | 31.188 | 0.070 |  | 0.000 | -0.088 | -5.395 | -5.921 |
| Nilslund1 | Octoploid | 39.286 | -0.820 |  | 0.000 | -0.409 | 3.860 | -2.807 |
| Nilslund2 | Octoploid | 37.731 | 0.060 |  | 0.000 | -0.329 | -5.029 | -7.105 |
| Norska1 | Octoploid | 57.500 | -0.328 | 2.128 | 0.000 | -0.175 | -5.351 | -7.412 |
| Rågången | Octoploid | 51.800 | 0.200 |  | -0.263 | 0.000 | 2.719 | 0.351 |
| Rågången2 | Octoploid | 52.500 | -0.610 |  | 0.000 | 0.000 | -5.877 | -5.877 |
| Svarv | Octoploid | 38.050 | -0.130 | 16.573 | 0.000 | -0.175 | -2.061 | -2.939 |
| V-m2 | Octoploid | 18.750 | -0.484 | -5.649 | 0.000 | 0.000 | -3.158 | -3.158 |
| Ö. Ämtvik2 | Octoploid | 21.500 | 0.050 |  | 0.000 | 0.000 | 1.930 | 1.930 |
| Bogslund | Tetraploid | 44.583 | 0.485 | -27.215 | 0.000 | -0.263 | -2.061 | -5.614 |
| Bölsäter2 | Tetraploid | 34.550 | 0.005 | -18.881 | 0.000 | -0.263 | -0.439 | -7.105 |
| Bölsäter3 | Tetraploid | 45.000 | 0.250 |  | 0.000 | -0.117 | -5.439 | -6.550 |
| Dagnäs1 | Tetraploid | 48.500 | -0.413 | 11.017 | 0.000 | -0.088 | -7.851 | -9.035 |
| Dagnäs2 | Tetraploid | 59.091 | -0.030 |  | 0.000 | 0.000 | -2.982 | -2.982 |
| Edeby1 | Tetraploid | 66.292 | 0.879 | 13.069 | 0.000 | -0.175 | -7.368 | -9.693 |
| Gravfältet | Tetraploid | 52.200 | 0.620 |  | 0.000 | 0.044 | -2.807 | -2.456 |
| Grinda | Tetraploid | 52.500 | -0.020 |  | 0.000 | -0.117 | 2.690 | 0.585 |
| Kallmyra1 | Tetraploid | 40.000 | -0.351 | 2.128 | 0.000 | 0.175 | -9.123 | -5.965 |
| Kallmyra2 | Tetraploid | 51.909 | -0.390 |  | 0.000 | -0.219 | -12.325 | -17.061 |
| Kallmyra3 | Tetraploid | 49.250 | 0.324 | -8.983 | 0.000 | -0.132 | -4.715 | -7.083 |
| Kohagen | Tetraploid | 36.750 | 0.050 |  | 0.000 | -0.307 | -0.994 | -6.798 |
| Kristinelund1 | Tetraploid | 23.667 | 0.410 |  | 0.000 | 0.000 | -18.070 | -18.070 |
| Kristinelund6 | Tetraploid | 69.167 | -0.660 |  | 0.000 | 0.000 | -3.860 | -3.860 |
| Larslund2 | Tetraploid | 36.545 | 0.130 |  | 0.000 | -0.088 | 1.009 | 0.263 |
| Larslund3 | Tetraploid | 47.708 | -0.200 |  | 0.000 | -0.117 | -6.491 | -8.772 |
| Norska2 | Tetraploid | 40.375 | 0.700 |  | 0.000 | -0.234 | -0.877 | -5.556 |
| Ryssinge1 | Tetraploid | 58.750 | 0.565 | -12.316 | 0.000 | -0.351 | 0.760 | -4.737 |
| Ryssinge2 | Tetraploid | 60.063 | 0.221 | 12.312 | 0.000 | -0.497 | -4.035 | -10.673 |
| Ryssinge3 | Tetraploid | 52.278 | -0.036 | -2.316 | 0.000 | -0.066 | -3.904 | -4.781 |
| Skogstorp 6ny | Tetraploid | 42.500 | 0.400 |  | 0.000 | -0.175 | 0.263 | -2.982 |
| Skogstorp1 | Tetraploid | 30.100 | 0.490 |  | 0.000 | -0.234 | -8.421 | -11.462 |
| Stene | Tetraploid | 47.583 | 0.390 |  | 0.000 | -0.263 | 0.263 | -3.684 |
| Vildhägn1 | Tetraploid | 17.583 | 0.470 |  | 0.000 | -0.526 | 1.842 | -8.509 |
| V-m1 | Tetraploid | 57.125 | 0.419 | -0.094 | 0.000 | -0.132 | -12.346 | -14.649 |

Each row represents one of 53 populations. The 21 rows with data on attack intensity represent the field populations in which the number of oviposited plants was recorded each flowering season during 2009-2012.
